# Peritonsillar Abscess Model for Ultrasound Diagnosis Using Inexpensive Materials

**DOI:** 10.21980/J86G9P

**Published:** 2020-01-15

**Authors:** Mustafa N Rasheed, Keel E Coleman, Timothy J Fortuna

**Affiliations:** * Virginia Tech Carilion School of Medicine, Department of Emergency Medicine, Roanoke, VA

## Abstract

**Audience:**

This low-cost peritonsillar abscess model is intended for the education of emergency medicine and otolaryngology residents and advanced care practitioners of all training levels.

**Introduction:**

With incidence rates as high as 124 per 100,000 in the 14–21 age range, peritonsillar abscesses (PTA) are one of the more common head and neck soft tissue infections encountered in the emergency department.^1^ Peritonsillar abscesses can present to the emergency department in critically ill patients with the dangers of airway compromise and further local spreading. Emergency medicine (EM) residents need practice to properly identify and to minimize procedural complications such as perforation of nearby vessels, aspiration pneumonitis, and airway compromise. A major tool used in the emergency department that can help prevent complications is the use of ultrasound, which the Accreditation Council for Graduate Medical Education (ACGME) requires residents to become proficient at.^2^ Historically, computed tomography (CT) scanning to diagnose along with blind drainage has been the method of choice. With a sensitivity of 95.2%, intraoral ultrasound can minimize both radiation and procedure related complications.^3^ The current simulators available come at significant capital expenditure and do not provide high-fidelity ultrasound experience. Here we design and implement a low-cost trainer for residents to use ultrasound to diagnose and drain a PTA.

**Educational Objectives:**

By the end of this instructional session learners should be able to: 1) identify and discuss the indications, contraindications, and complications associated with peritonsillar abscesses, 2) properly identify and measure a PTA through ultrasound, and 3) competently perform ultrasound-guided peritonsillar abscess drainage on a simulator and remove fluid.

**Educational Methods:**

This PTA model utilizes task trainers designed from Styrofoam wig heads. An airway was modeled using readily available wood shop tools and balloons filled with a fluid mixture containing coconut lotion, water, and fragrance beads, which were inserted into the airway. This unique mixture within the balloons creates a realistic echogenicity of an abscess with loculations. With emergency medicine clinical faculty guidance and the use of ultrasound, learners are able to identify a peritonsillar abscess and subsequently demonstrate drainage of fluid with a needle and syringe.

**Research Methods:**

This PTA model was tested with a group of 36 emergency medicine residents. Optional, anonymous post surveys were completed by 17 residents. A 5-point Likert Scale was used to assess utility of this model.

**Results:**

The majority of users agreed the model provides a realistic image of the disease for diagnosis by ultrasound with a score of 3.6 and felt more comfortable identifying and draining peritonsillar abscesses with scores of 3.7 and 3.6 respectively. Learners’ surveys revealed the session was useful and improved their knowledge with both scoring 3.8. No critical feedback was given by learners or instructors. The efficacy of the content was assessed by evaluators observing proper ultrasound, procedure set up, and drainage of PTA.

**Discussion:**

This inexpensive model to expose residents to proper PTA drainage was effective considering learners’ high response to post-procedure survey scales. The results of our pilot implementation showed this model has utility in teaching ultrasound guided identification and drainage of PTA’s. With minimal build and optimized instruction time, we can improve residents’ comfort in performing this procedure and allow for important simulation experience in a safe, controlled environment.

**Topics:**

Simulation, emergency medicine, peritonsillar abscess, otolaryngology.

## USER GUIDE

[Table t1-jetem-5-1-i1]

**Table t1-jetem-5-1-i1:** 

List of Resources:	
Abstract	1
User Guide	3
Instructor Materials	8

**Learner Audience:**

Medical Students, Interns, Junior Residents, Senior Residents, Otolaryngology Residents, Attending Physicians, Advanced Clinical Practitioners

**Time Required for Implementation:**

**Preparation:** We approximate 15 minutes per simulation model to gather supplies, create airway, and insert abscess.

**Didactics:** Presenter should use about 15 minutes to present the model and use it as a tool to describe set up, methods to ensure successful identification and aspiration, and possible complications. Learners will use a total of 15 minutes to describe the procedure, ultrasound, and practice the procedure on the model. There will be a 5 minute debrief post-procedure to discuss if there were any issues with procedure and simulator and ways to improve.

**Recommended Number of Learners per Instructor:**

The ratio of learners to instructors should not exceed 6:1. Pairs of students may alternate between assisting with supplies and performing the procedure with the use of ultrasound.

**Topics:**

Simulation, emergency medicine, peritonsillar abscess, otolaryngology.

**Objectives:**

By the end of this instructional session learners should be able to:

1. Identify and discuss the indications, contraindications, and complications associated with peritonsillar abscesses.
2. Properly identify and measure a PTA through ultrasound.
3. Competently perform ultrasound-guided peritonsillar abscess drainage on a simulator and remove fluid.

### Linked objectives and methods

We recommend an interactive didactic session followed by a hands-on experience. This allows for learners to discuss the natural history of peritonsillar abscesses as well as the key features of the aspiration procedure to treat peritonsillar abscesses (objective 1). Learners are then able to immediately apply the newfound knowledge as they use an ultrasound to identify a peritonsillar abscess on the model and subsequently aspirate the modeled abscess (objectives 2 and 3).

### Recommended pre-reading for instructor

- Riviello RJ. Otolaryngologic procedures; tonsil: peritonsillar abscess. In: Roberts JR, ed. *Roberts and Hedges’ Clinical Procedures in Emergency Medicine*. 7th ed. Philadelphia, PA: Elsevier/Saunders; 2019: 1338–1383.

### Learner responsible content (LRC)

- Coneybeare D. Peritonsillar abscess. Core EM Podcast. https://coreem.net/core/peritonsillar-abscess/. Published June 9, 2015. Accessed August 5, 2019.
- Riviello RJ. Otolaryngologic procedures; tonsil: peritonsillar abscess. In: Roberts JR, ed. *Roberts and Hedges’ Clinical Procedures in Emergency Medicine*. 7th ed. Philadelphia, PA: Elsevier/Saunders; 2019: 1338–1383.
- Wald ER. Peritonsillar cellulitis and abscess. In: Wiley JF, ed. *UpToDate*. Waltham, MA: UpToDate, Inc. https://www.uptodate.com/contents/peritonsillarcellulitis-and-abscess. Updated November 15, 2017. Accessed May 13, 2019.

### Associated content

- Peritonsillar Abscess for the Emergency Physician PowerPoint
- Peritonsillar Abscess Ultrasound Evaluation

### Implementation Methods

The instructional session should first begin with a brief didactic lesson that covers the necessary knowledge to perform a peritonsillar abscess aspiration. This includes causes and formation of the abscess, critical anatomy, tools required for aspiration, ultrasound, and the method of aspiration. The educator should also discuss how the proper use of ultrasound and procedure set up can limit complications such as major vessel injury and airway compromise. Ideally, the educator will utilize the model throughout the presentation. Following this, students will form pairs and walk through the process of performing a peritonsillar aspiration with the supervision of instructors.

### List of items required to replicate this innovation

1. Styrofoam Wig Head $2.43
2. Balloons $2.99 for 15ct
3. Fragrance Beads $3.97 for 12 oz
4. Coconut Lotion $5.00 for 14 oz
5. 20–22G needle or spinal needle with cap
6. 10 cc syringe
7. Mobile ultrasound with gynecology mode and 5–10mHz curved array endocavitary probe
8. Macintosh blades and handle
9. Local Anesthetic: nebulized lidocaine, benzocaine spray, or similar substitute

### Options for purchasing

1. Wig Head $2.43, Amazon (https://www.amazon.com/gp/product/B001BAN0FK/ref=ox_sc_act_title_1?smid=A3Q9UMQDRTL5UQ&psc=1
2. Gonzo Natural Magic Fragrance Beads - Odor Absorbing - 12 Ounce, Amazon https://www.amazon.com/Gonzo-Natural-Magic-Fragrance-Beads/dp/B00U2MEQZQ/ref=sr_1_21?keywords=odor+Gel+Beads&qid=1556583620&s=gateway&sr=8-21
3. Assorted Color Balloons 15ct 12in Latex Balloons, PartyCity https://www.partycity.com/assorted-color-balloons-15ct-237864.html?gclid=CjwKCAjwqqrmBRAAEiwAdpDXtOfuYIjJtAHfVX6a-RCHbKqtv8PzIklqvp5nxbqM-coje4lb8qef8xoCWuQQAvD_BwE&gclsrc=aw.ds&extcmp=pla%7CGoogle
4. Coconut Oil Body Lotion 13.5 fl. oz, Amazon https://www.amazon.com/Palmers-Coconut-Formula-Lotion-Bottle/dp/B015ORMEP2/ref=sr_1_2?crid=XM284VALQ83F&keywords=coconut+lotion&qid=1556842967&refinements=p_85%3A2470955011&rnid=2470954011&rps=1&s=gateway&sprefix=coconut+lo%2Caps%2C130&sr=8-2

### Approximate cost of items to create this innovation

$15

### Detailed methods to construct this innovation

1. The PTA is assembled by initially establishing an airway in the wig head. Using a wood drill bit, start on the lateral aspect of the model head under the ears and drill until the opening is about 2.5 cm in diameter. Create a similar opening where the mouth of the model is. This will form the airway and allow access to insert the abscess.[Fig f1-jetem-5-1-i1]
  **Figure f1-jetem-5-1-i1:**
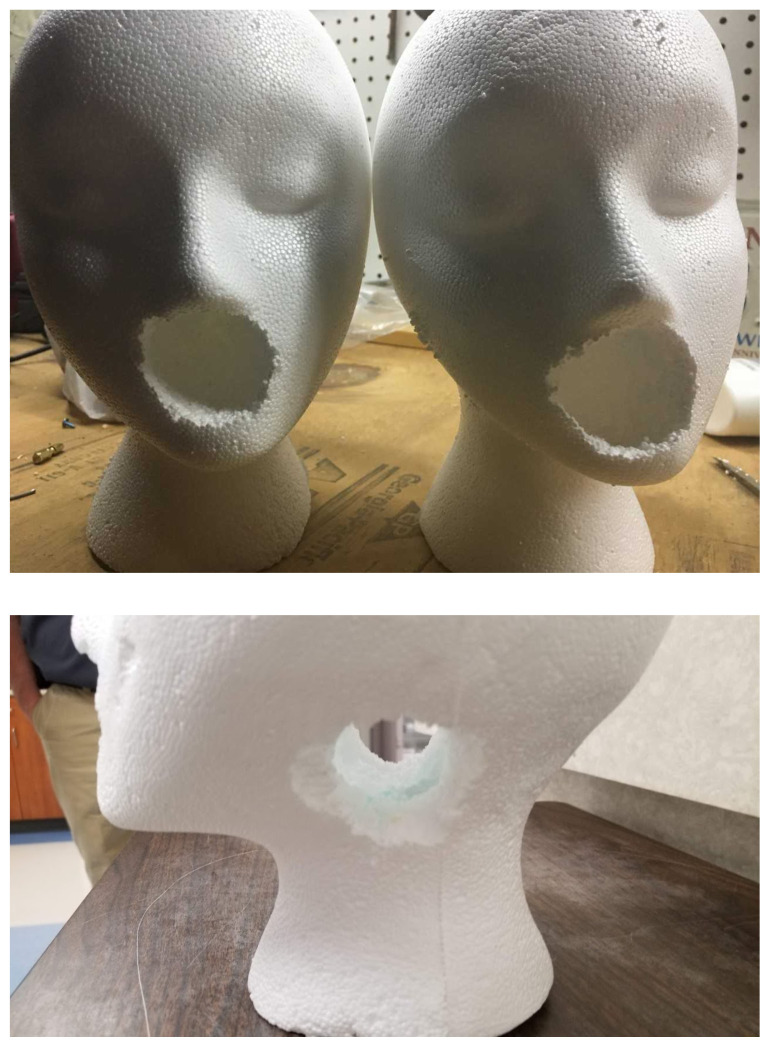
Anterior and lateral view.
2. Insert 5–10 fragrance beads into a balloon, followed by 1:2 ratio of coconut oil to water until the abscess model is the desired size. We recommend a diameter of roughly 2 cm to fit properly in the airway and prevent the abscess model from dislodging.[Fig f2-jetem-5-1-i1]
  **Figure f2-jetem-5-1-i1:**
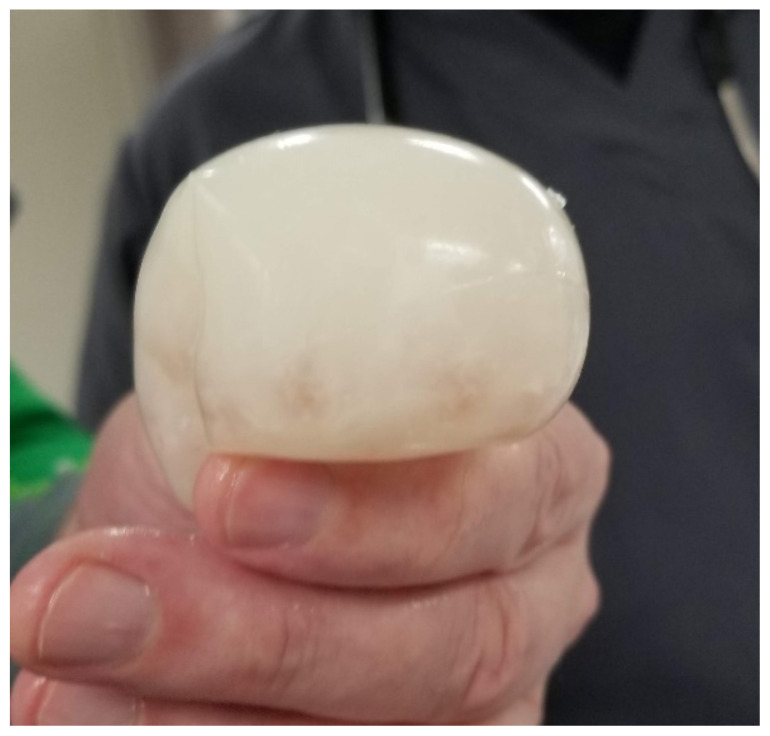
Abscess.
3. Insert the abscess in the airway in the region near suspected tonsils and the model is complete. The abscess model should be able to sit snuggly in the airway since the model is not hollow below the oral cavity.[Fig f3-jetem-5-1-i1]
  **Figure f3-jetem-5-1-i1:**
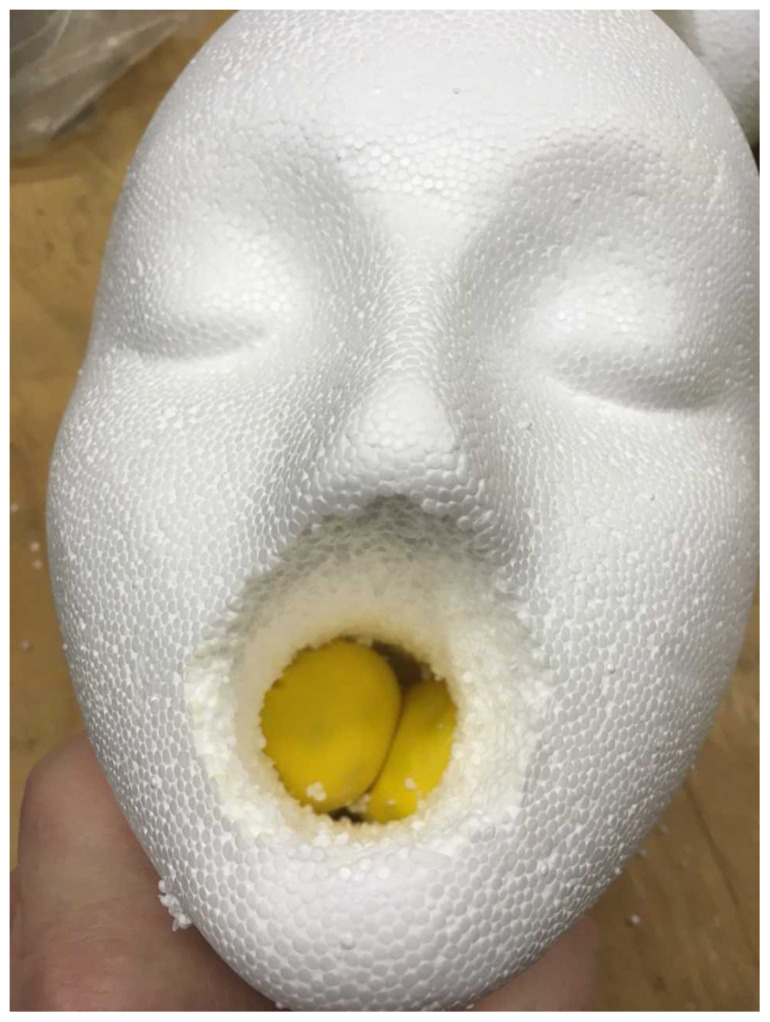
Peritonsillar Abscess.

The simulation is complete. A checklist is provided for evaluators to use for guidance and ensure a standardized experience. Learners may identify and measure the PTA with ultrasound using the intracavitary probe.[Fig f4-jetem-5-1-i1][Fig f5-jetem-5-1-i1][Fig f6-jetem-5-1-i1][Fig f7-jetem-5-1-i1]

**Figure f4-jetem-5-1-i1:**
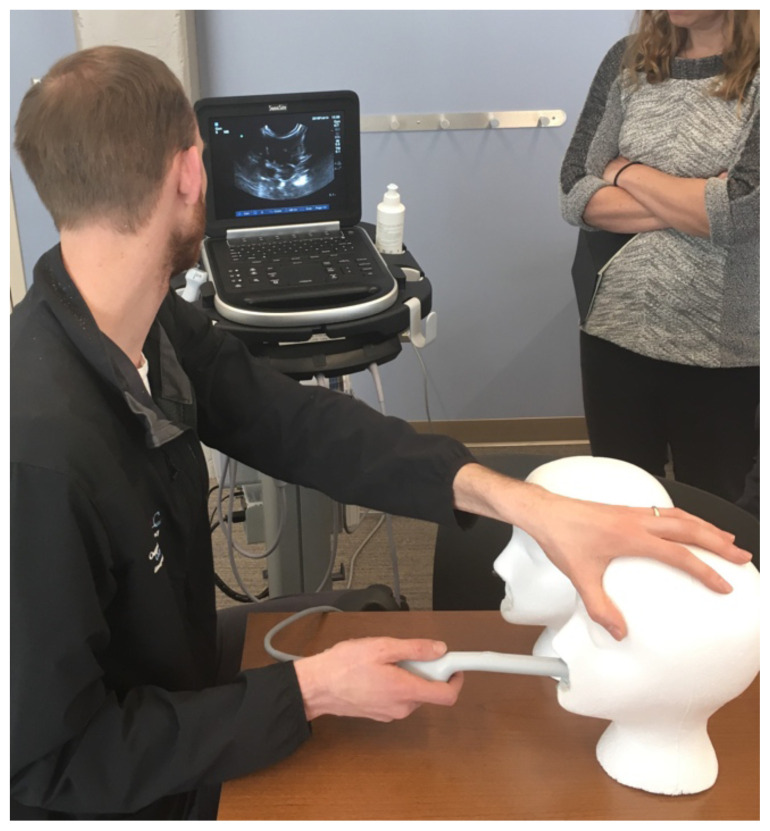
Intracavitary probe use.

**Figure f5-jetem-5-1-i1:**
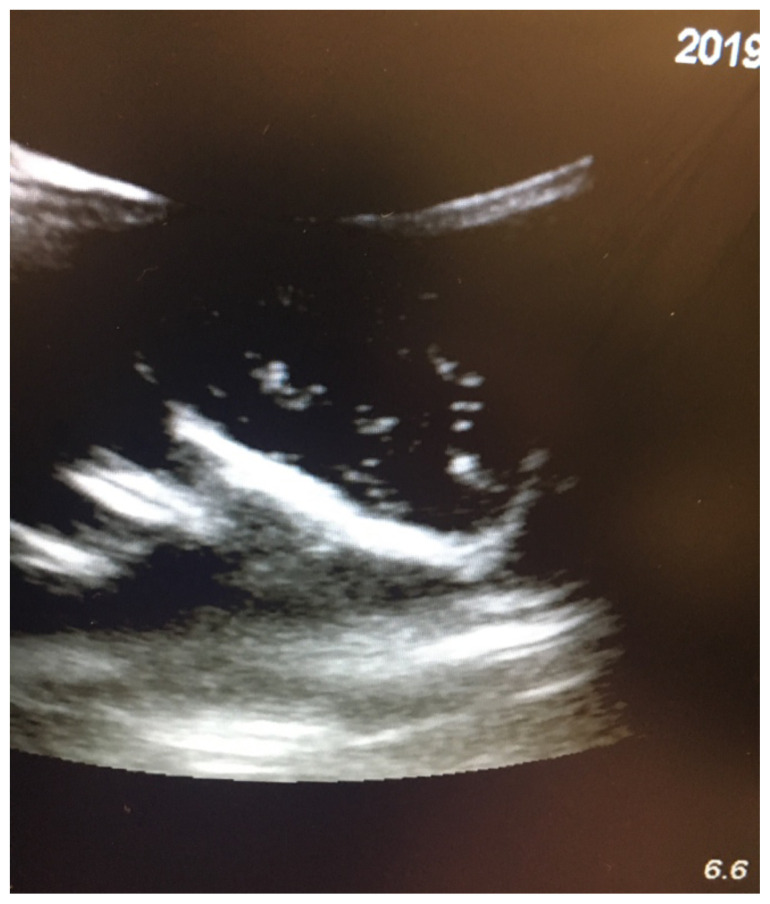
Model PTA Ultrasound.

**Figure f6-jetem-5-1-i1:**
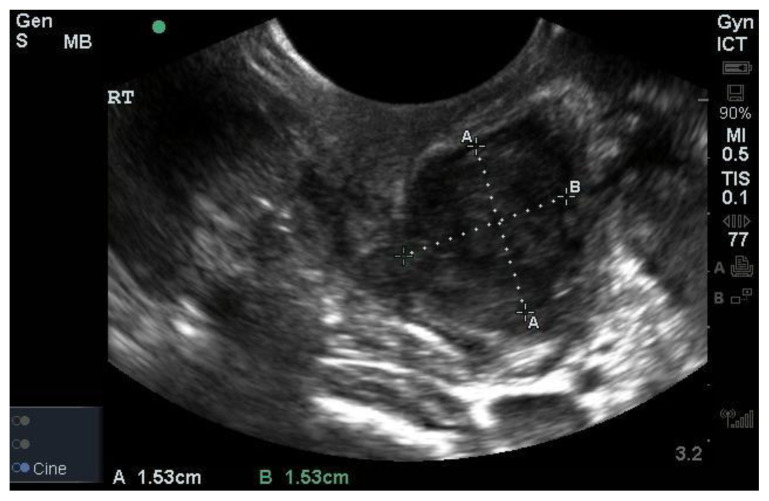
Actual Peritonsillar Abscesses for comparison. Photo courtesy of Dr. John Nogueira, Carilion Clinic Emergency Medicine.

After proper set up and discussion of the procedure, learners may drain the PTA with an appropriately sized syringe and needle.

**Figure f7-jetem-5-1-i1:**
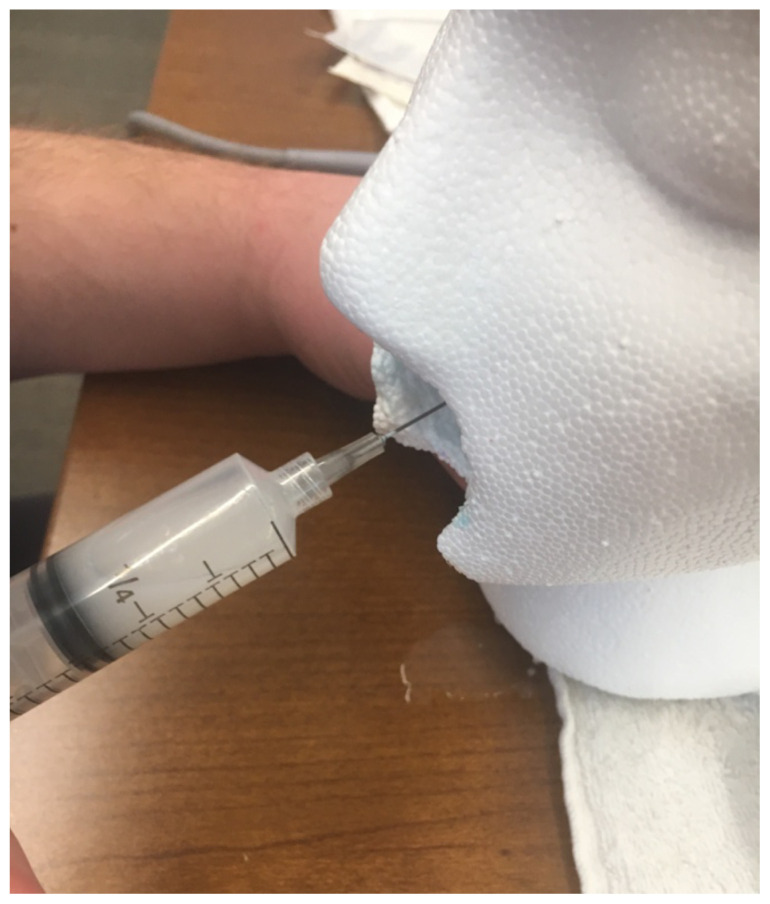
PTA drainage.

### Results and tips for successful implementation

In order to reiterate critical anatomy, have learners measure abscess size and determine the needle depth required to avoid posterior structures. Students may cut needle caps with trauma shears to assist with aspiration and limit complications. In addition, it is important to address patient position, comfort, and tools to maximize airway visibility such as with the use of a tongue depressors or a Mac blade. This PTA model was tested with a group of 36 emergency medicine residents. Optional, anonymous post surveys were completed by 17 residents. A 5-question 5-point Likert Scale was used to assess utility of this model with room for general feedback. The majority of users agreed the model provides a realistic image of the disease for diagnosis by ultrasound and felt more comfortable identifying a peritonsillar abscess with scores of 3.6 and 3.7 on the Likert scale. Learners’ surveys revealed the session was useful and improved their knowledge with both receiving scores of 3.8. No critical feedback was given by learners or instructors.

## Figures and Tables

**Table t2-jetem-5-1-i1:** 

	Date_________
Name________________________	Evaluator________________________

**Table t3-jetem-5-1-i1:** 

Describe ideal position of patient.	
Seated with emesis basin nearby.	

Select proper ultrasound probe and proper preparation to perform ultrasound.	
5-10mHz curved array endocavitary probe should be used with probe cover. May discuss the use of small linear probe or transcutaneous approach under the mandible.	

Discuss methods to increase patient comfort and abscess visibility.	
Patient should be seated. Mac blade or tongue depressor can be used to increase visibility. Local Anesthetic: nebulized lidocaine, benzocaine spray, or injected anesthetics to decrease pain.	

Ultrasound and measure size of abscess.	
Learner should describe area of interest, select proper gain and depth of the abscess and measure it in at least 2 planes.	

Preparation for drainage with needle cap cut to proper length.	

Proper drainage of abscess.	

**Pass/Fail** (>80%)	

Comments:	

## INSTRUCTOR MATERIALS

### Peritonsillar Abscess for the Emergency Physician PowerPoint

Please see associated lecture

### Peritonsillar Ultrasound Model Evaluation

[Table t2-jetem-5-1-i1] [Table t3-jetem-5-1-i1]
