# Supplementary material for: Peritonsillar Abscess Model for Ultrasound Diagnosis Using Inexpensive Materials
Source: J Educ Teach Emerg Med. 2020 Jan 15;5(1):I1–9. doi: 10.21980/J86G9P (PMC10332534; doi:10.21980/J86G9P)
Supplement: Supplementary file 1 — Please see associated lecture [file jetem-5-1-i1-supp1.pptx]

## Slide 1
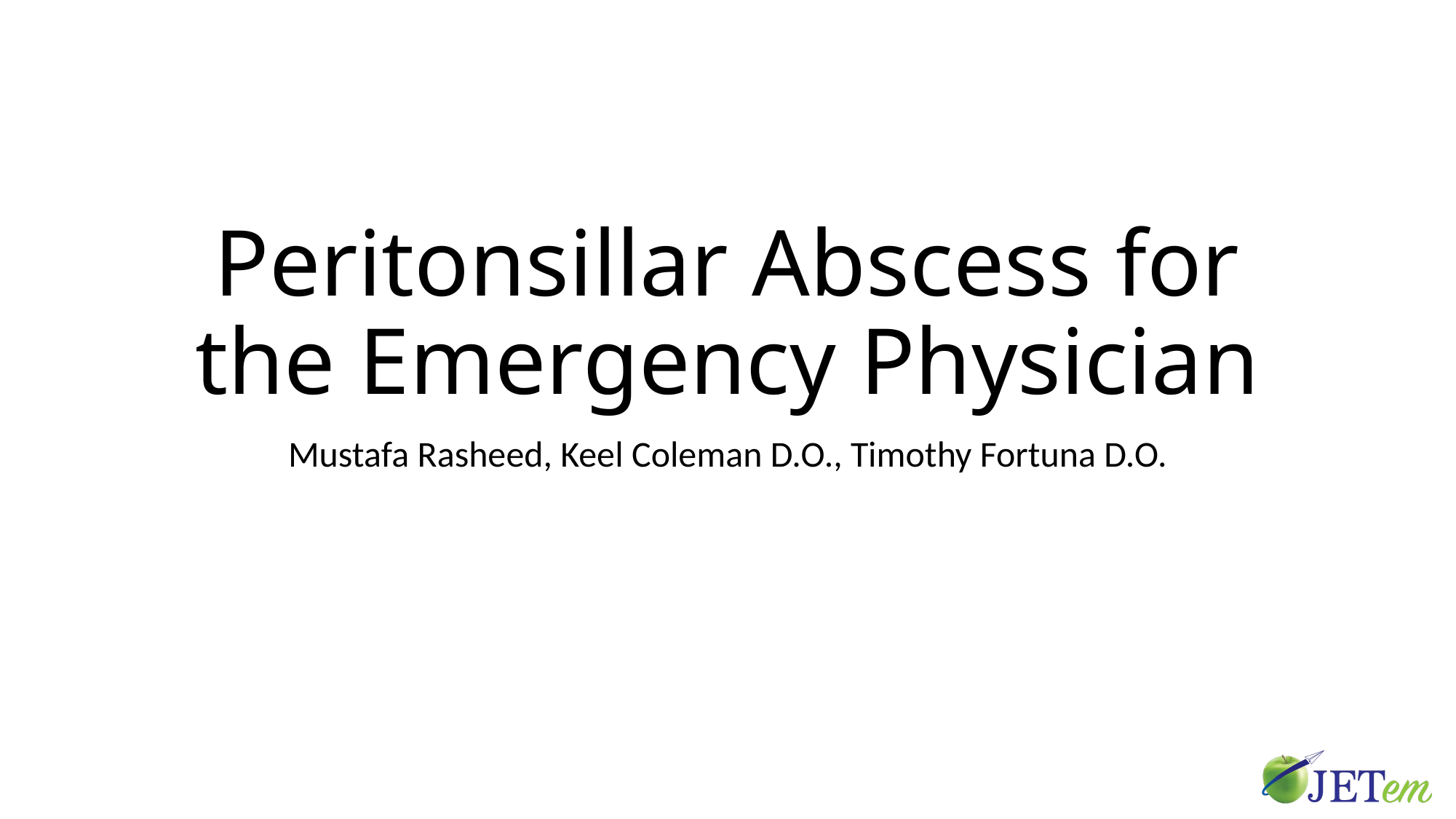

# Peritonsillar Abscess for the Emergency Physician
Mustafa Rasheed, Keel Coleman D.O., Timothy Fortuna D.O.

## Slide 2
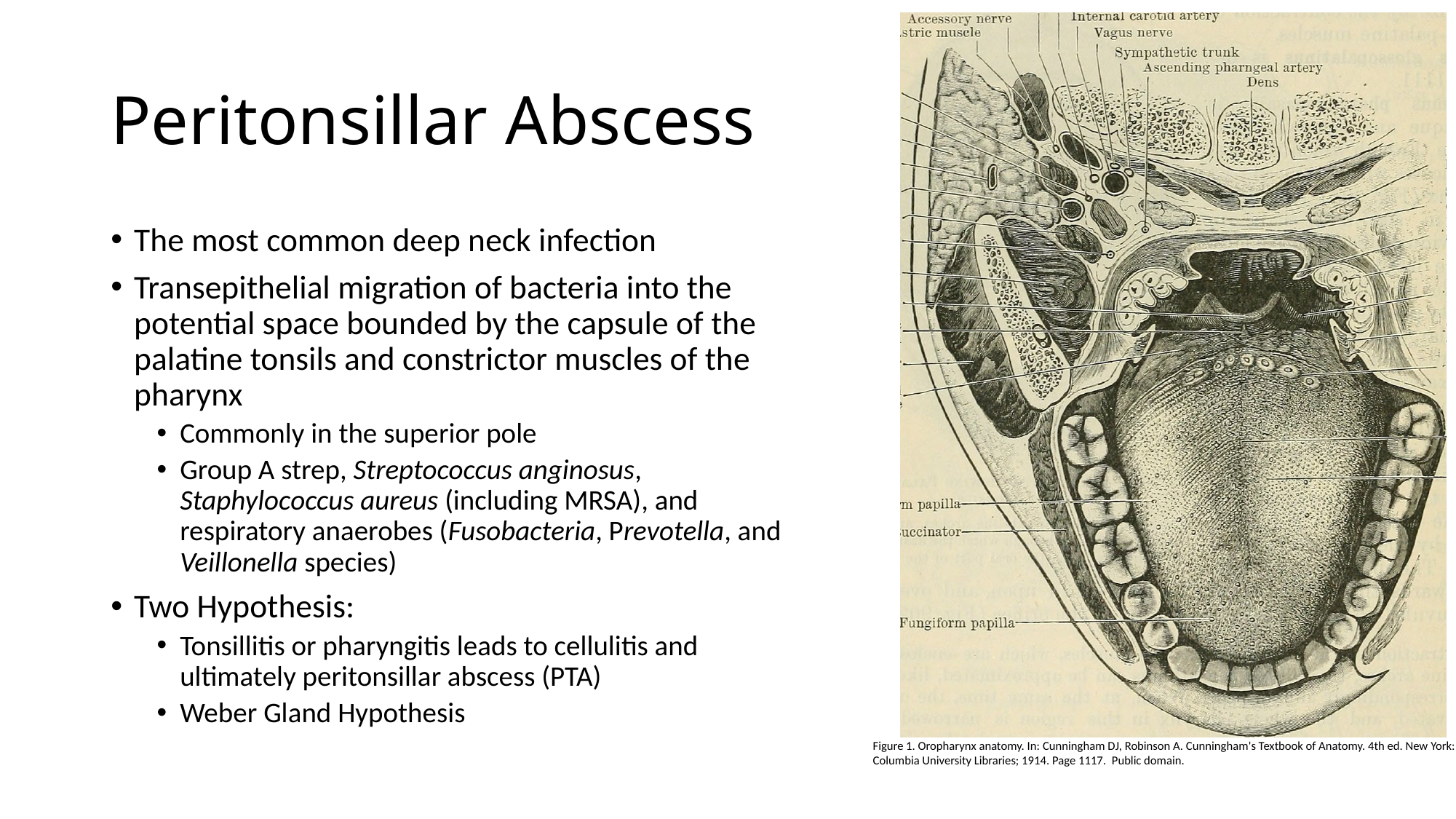

# Peritonsillar Abscess
The most common deep neck infection
Transepithelial migration of bacteria into the potential space bounded by the capsule of the palatine tonsils and constrictor muscles of the pharynx
Commonly in the superior pole
Group A strep, Streptococcus anginosus, Staphylococcus aureus (including MRSA), and respiratory anaerobes (Fusobacteria, Prevotella, and Veillonella species)
Two Hypothesis:
Tonsillitis or pharyngitis leads to cellulitis and ultimately peritonsillar abscess (PTA)
Weber Gland Hypothesis
Figure 1. Oropharynx anatomy. In: Cunningham DJ, Robinson A. Cunningham's Textbook of Anatomy. 4th ed. New York: Columbia University Libraries; 1914. Page 1117. Public domain.

## Slide 3
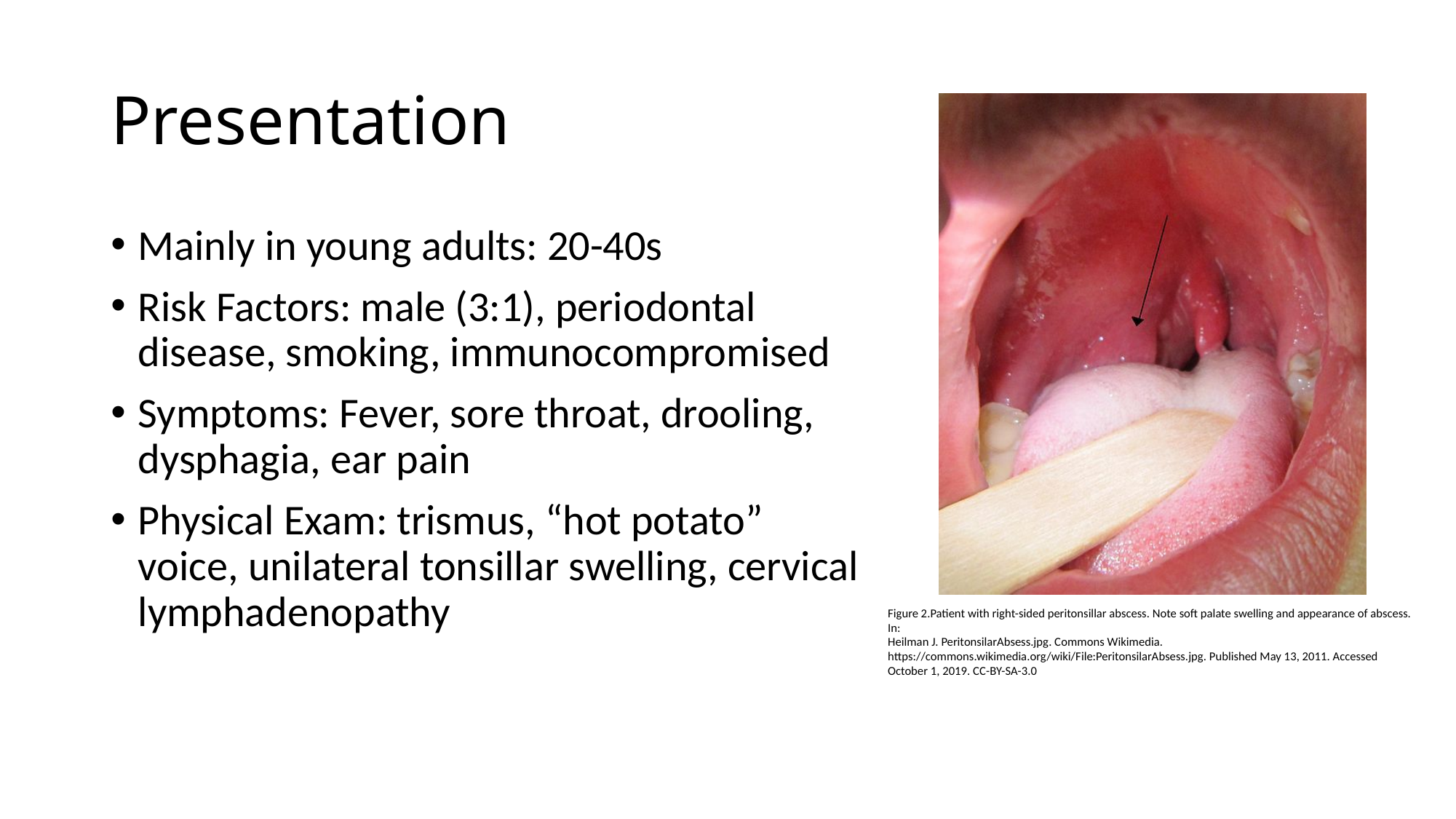

# Presentation
Mainly in young adults: 20-40s
Risk Factors: male (3:1), periodontal disease, smoking, immunocompromised
Symptoms: Fever, sore throat, drooling, dysphagia, ear pain
Physical Exam: trismus, “hot potato” voice, unilateral tonsillar swelling, cervical lymphadenopathy
Figure 2.Patient with right-sided peritonsillar abscess. Note soft palate swelling and appearance of abscess. In:
Heilman J. PeritonsilarAbsess.jpg. Commons Wikimedia. https://commons.wikimedia.org/wiki/File:PeritonsilarAbsess.jpg. Published May 13, 2011. Accessed October 1, 2019. CC-BY-SA-3.0

## Slide 4
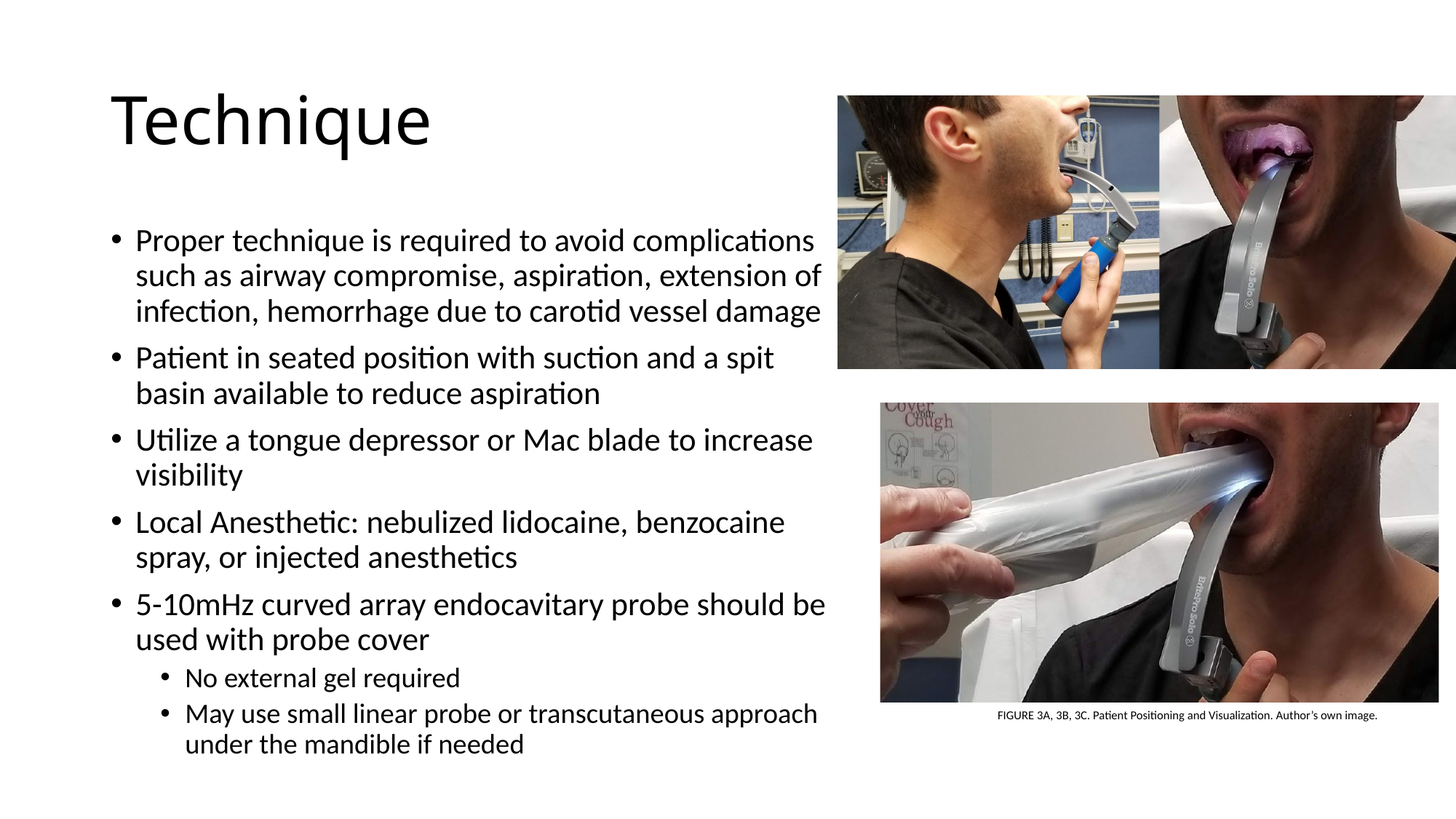

# Technique
Proper technique is required to avoid complications such as airway compromise, aspiration, extension of infection, hemorrhage due to carotid vessel damage
Patient in seated position with suction and a spit basin available to reduce aspiration
Utilize a tongue depressor or Mac blade to increase visibility
Local Anesthetic: nebulized lidocaine, benzocaine spray, or injected anesthetics
5-10mHz curved array endocavitary probe should be used with probe cover
No external gel required
May use small linear probe or transcutaneous approach under the mandible if needed
FIGURE 3A, 3B, 3C. Patient Positioning and Visualization. Author’s own image.

## Slide 5
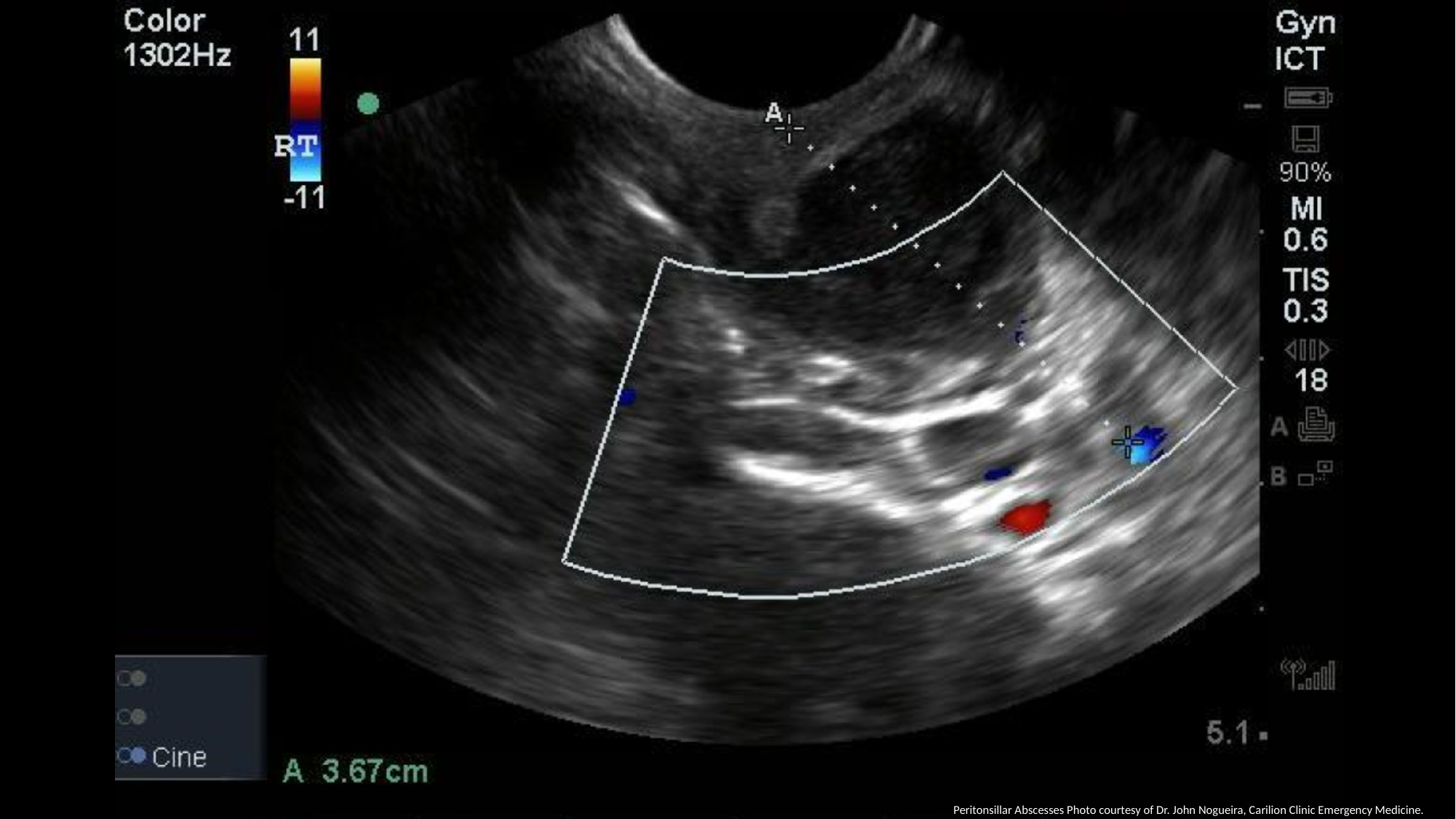

Peritonsillar Abscesses Photo courtesy of Dr. John Nogueira, Carilion Clinic Emergency Medicine.

## Slide 6
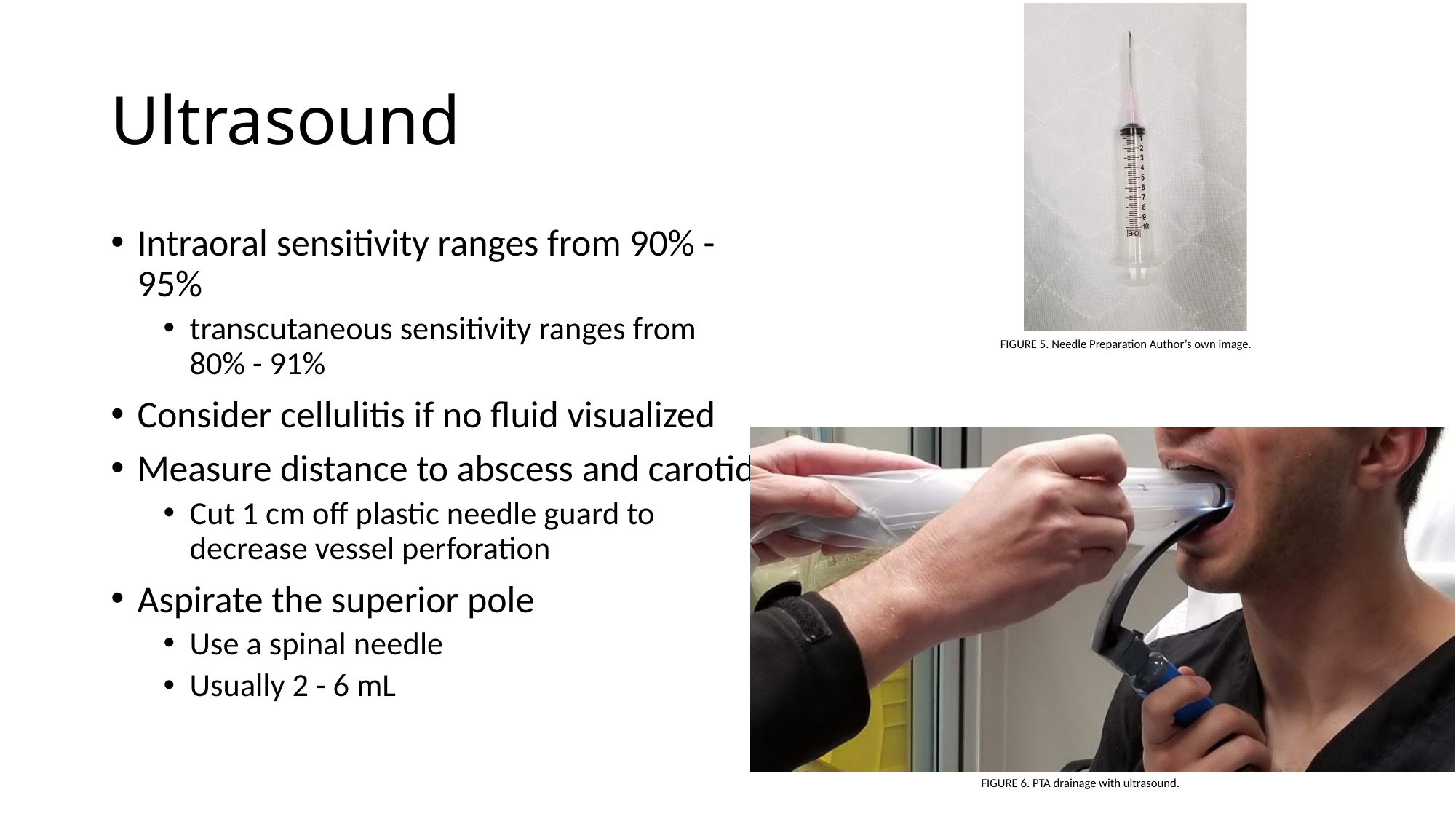

# Ultrasound
Intraoral sensitivity ranges from 90% -95%
transcutaneous sensitivity ranges from 80% - 91%
Consider cellulitis if no fluid visualized
Measure distance to abscess and carotid
Cut 1 cm off plastic needle guard to decrease vessel perforation
Aspirate the superior pole
Use a spinal needle
Usually 2 - 6 mL
FIGURE 5. Needle Preparation Author’s own image.
FIGURE 6. PTA drainage with ultrasound.

## Slide 7
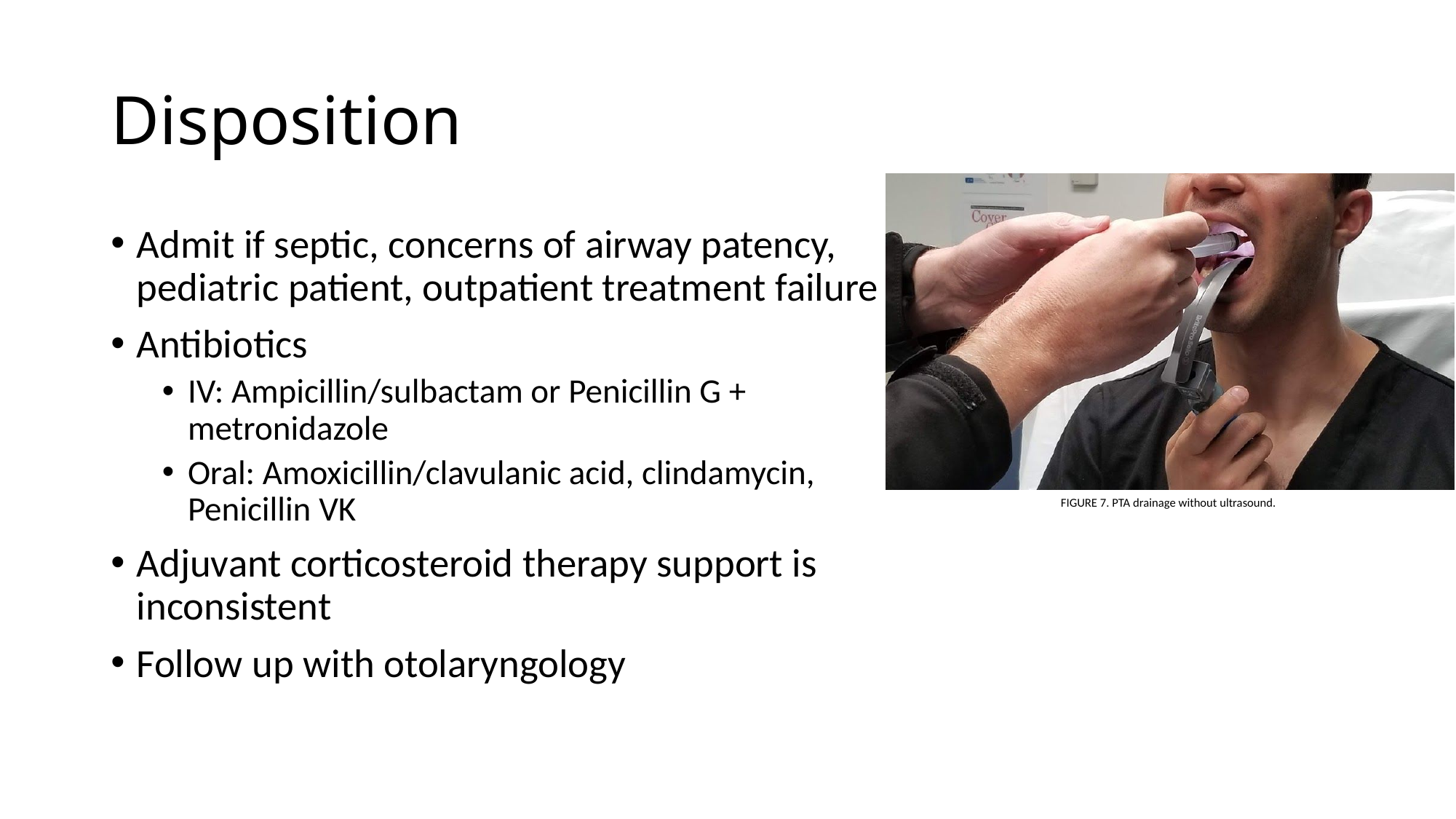

# Disposition
Admit if septic, concerns of airway patency, pediatric patient, outpatient treatment failure
Antibiotics
IV: Ampicillin/sulbactam or Penicillin G + metronidazole
Oral: Amoxicillin/clavulanic acid, clindamycin, Penicillin VK
Adjuvant corticosteroid therapy support is inconsistent
Follow up with otolaryngology
FIGURE 7. PTA drainage without ultrasound.

## Slide 8
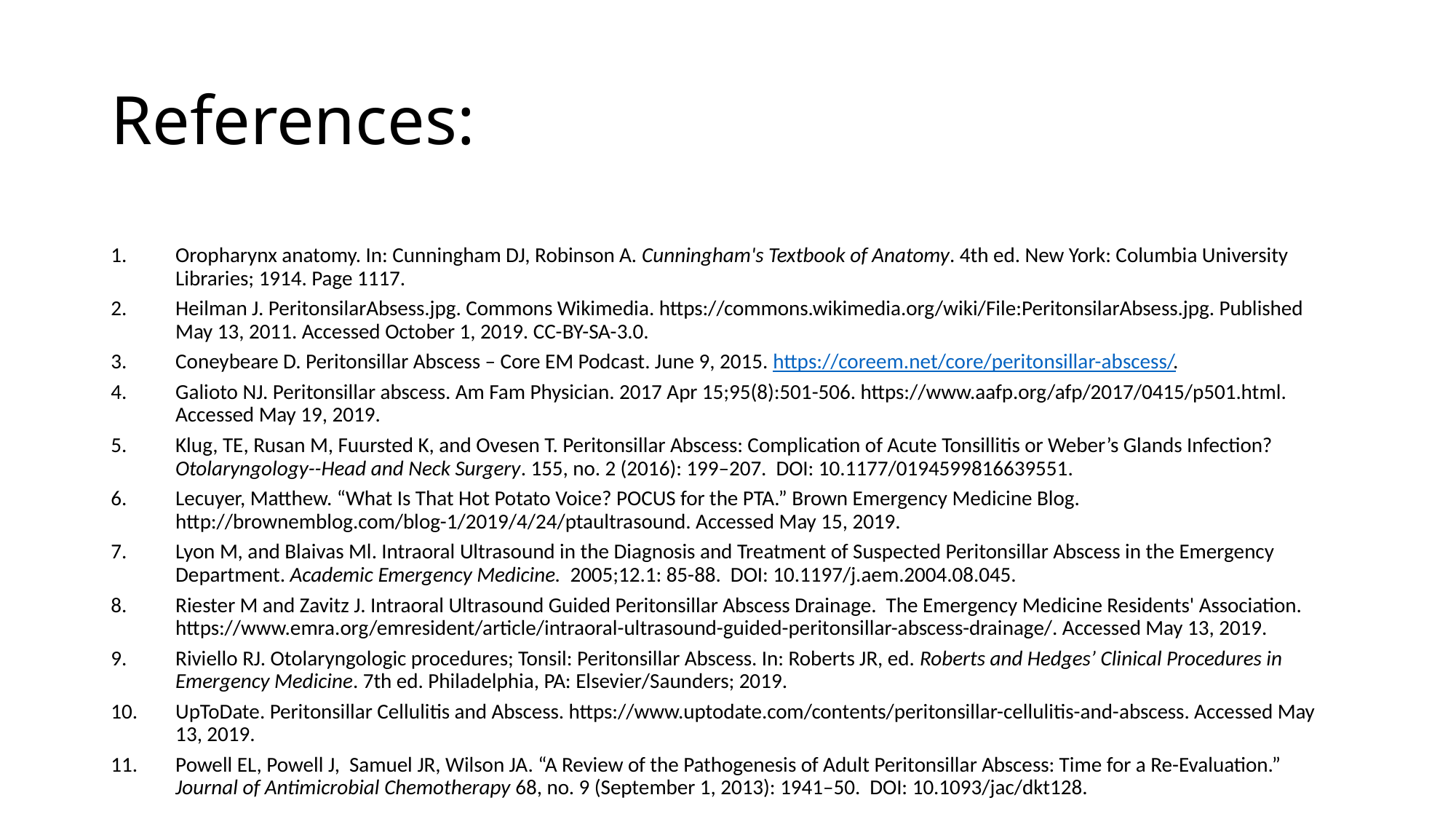

# References:
Oropharynx anatomy. In: Cunningham DJ, Robinson A. Cunningham's Textbook of Anatomy. 4th ed. New York: Columbia University Libraries; 1914. Page 1117.
Heilman J. PeritonsilarAbsess.jpg. Commons Wikimedia. https://commons.wikimedia.org/wiki/File:PeritonsilarAbsess.jpg. Published May 13, 2011. Accessed October 1, 2019. CC-BY-SA-3.0.
Coneybeare D. Peritonsillar Abscess – Core EM Podcast. June 9, 2015. https://coreem.net/core/peritonsillar-abscess/.
Galioto NJ. Peritonsillar abscess. Am Fam Physician. 2017 Apr 15;95(8):501-506. https://www.aafp.org/afp/2017/0415/p501.html. Accessed May 19, 2019.
Klug, TE, Rusan M, Fuursted K, and Ovesen T. Peritonsillar Abscess: Complication of Acute Tonsillitis or Weber’s Glands Infection? Otolaryngology--Head and Neck Surgery. 155, no. 2 (2016): 199–207. DOI: 10.1177/0194599816639551.
Lecuyer, Matthew. “What Is That Hot Potato Voice? POCUS for the PTA.” Brown Emergency Medicine Blog. http://brownemblog.com/blog-1/2019/4/24/ptaultrasound. Accessed May 15, 2019.
Lyon M, and Blaivas Ml. Intraoral Ultrasound in the Diagnosis and Treatment of Suspected Peritonsillar Abscess in the Emergency Department. Academic Emergency Medicine. 2005;12.1: 85-88. DOI: 10.1197/j.aem.2004.08.045.
Riester M and Zavitz J. Intraoral Ultrasound Guided Peritonsillar Abscess Drainage. The Emergency Medicine Residents' Association. https://www.emra.org/emresident/article/intraoral-ultrasound-guided-peritonsillar-abscess-drainage/. Accessed May 13, 2019.
Riviello RJ. Otolaryngologic procedures; Tonsil: Peritonsillar Abscess. In: Roberts JR, ed. Roberts and Hedges’ Clinical Procedures in Emergency Medicine. 7th ed. Philadelphia, PA: Elsevier/Saunders; 2019.
UpToDate. Peritonsillar Cellulitis and Abscess. https://www.uptodate.com/contents/peritonsillar-cellulitis-and-abscess. Accessed May 13, 2019.
Powell EL, Powell J, Samuel JR, Wilson JA. “A Review of the Pathogenesis of Adult Peritonsillar Abscess: Time for a Re-Evaluation.” Journal of Antimicrobial Chemotherapy 68, no. 9 (September 1, 2013): 1941–50. DOI: 10.1093/jac/dkt128.
